# Exposure to arsenic in drinking water is associated with increased prevalence of diabetes: a cross-sectional study in the Zimapán and Lagunera regions in Mexico

**DOI:** 10.1186/1476-069X-10-73

**Published:** 2011-08-24

**Authors:** Luz M Del Razo, Gonzalo G García-Vargas, Olga L Valenzuela, Erika Hernández Castellanos, Luz C Sánchez-Peña, Jenna M Currier, Zuzana Drobná, Dana Loomis, Miroslav Stýblo

**Affiliations:** 1 Departamento de Toxicología, Centro de Investigación y de Estudios Avanzados del Instituto Politécnico Nacional, México DF, México; 2 Facultad de Medicina, Universidad Juárez del Estado de Durango, Gómez Palacio, Durango, México; 3 Curriculum in Toxicology, University of North Carolina at Chapel Hill, Chapel Hill, North Carolina, USA; 4 Department of Nutrition, University of North Carolina at Chapel Hill, Chapel Hill, North Carolina, USA; 5 School of Community Health Sciences/MS-274, University of Nevada, Reno, Nevada, USA (Current affiliation: Department of Epidemiology and Eppley Cancer Institute, University of Nebraska Medical Center, Omaha NE, USA

**Keywords:** Arsenic, drinking water, diabetes, urinary metabolites of arsenic, dimethylarsinite

## Abstract

**Background:**

Human exposures to inorganic arsenic (iAs) have been linked to an increased risk of diabetes mellitus. Recent laboratory studies showed that methylated trivalent metabolites of iAs may play key roles in the diabetogenic effects of iAs. Our study examined associations between chronic exposure to iAs in drinking water, metabolism of iAs, and prevalence of diabetes in arsenicosis-endemic areas of Mexico.

**Methods:**

We used fasting blood glucose (FBG), fasting plasma insulin (FPI), oral glucose tolerance test (OGTT), glycated hemoglobin (HbA1c), and insulin resistance (HOMA-IR) to characterize diabetic individuals. Arsenic levels in drinking water and urine were determined to estimate exposure to iAs. Urinary concentrations of iAs and its trivalent and pentavalent methylated metabolites were measured to assess iAs metabolism. Associations between diabetes and iAs exposure or urinary metabolites of iAs were estimated by logistic regression with adjustment for age, sex, hypertension and obesity.

**Results:**

The prevalence of diabetes was positively associated with iAs in drinking water (OR 1.13 per 10 ppb, p < 0.01) and with the concentration of dimethylarsinite (DMAs^III^) in urine (OR 1.24 per inter-quartile range, p = 0.05). Notably, FPI and HOMA-IR were negatively associated with iAs exposure (β -2.08 and -1.64, respectively, p < 0.01), suggesting that the mechanisms of iAs-induced diabetes differ from those underlying type-2 diabetes, which is typically characterized by insulin resistance.

**Conclusions:**

Our study confirms a previously reported, but frequently questioned, association between exposure to iAs and diabetes, and is the first to link the risk of diabetes to the production of one of the most toxic metabolites of iAs, DMAs^III^.

## Background

Results of several epidemiologic studies have suggested that chronic environmental or occupational exposures to inorganic arsenic (iAs) increase risk of diabetes. These studies, as well as, other studies examining the diabetogenic effects of iAs exposure were recently reviewed by Navas-Acien and coworkers [1]. The association between iAs exposure and diabetes has been frequently questioned because of methodological problems associated with some of these studies and because these studies focused mainly on populations exposed to high levels of iAs [1]. In addition, results of studies examining effects of low exposures to iAs did not support this association or yielded conflicting results [1]. For example, a recent study utilizing data collected by the National Health and Nutrition Examination Survey found a significant association between urinary arsenic and diabetes in the general U.S. population [2]. Another U.S. study found significant associations between cumulative exposure to iAs and risk of depression or high blood pressure, but not diabetes [3]. Conflicting information has also been provided by studies that examined the diabetogenic effects of iAs in laboratory animals. These studies were recently reviewed [1,4]. In contrast, research using *ex vivo *or tissue culture models has consistently shown that exposures to subtoxic concentrations of trivalent iAs or trivalent methylated arsenicals produce effects consistent with diabetes, including inhibition of insulin production by pancreatic β-cells and inhibition of basal or insulin-stimulated glucose uptake by skeletal muscle, cultured adipocytes, or kidney cells [4]. The tissue culture studies have also provided clues about molecular mechanisms of the diabetogenic effects of iAs exposure and have highlighted the role of specific metabolites of iAs in these mechanisms.

The pathway for iAs metabolism in humans involves the reduction of As^V^-species to As^III^-species followed by oxidative methylation of As^III^-species, yielding mono- and dimethylated metabolites that contain either As^III ^or As^V ^[5]. Arsenic (+3 oxidation state) methyltransferase (AS3MT) is the key enzyme in this pathway [5,6]. The trivalent iAs, arsenite (iAs^III^), and its methylated metabolites containing trivalent As, methylarsonite (MAs^III^) and dimethylarsinite (DMAs^III^), are generally more toxic and reactive than their pentavalent counterparts, arsenate (iAs^V^), methylarsonate (MAs^V^) and dimethylarsinate (DMAs^V^) [7]. These trivalent arsenicals are also potent inhibitors of insulin-stimulated glucose uptake by cultured murine adipocytes [8,9]. Notably, MAs^III ^and DMAs^III ^are more potent than iAs^III ^as inhibitors of key steps in the insulin-activated signal transduction pathway, specifically, the insulin-dependent phosphorylation of protein kinase-B (PKB) or PKB-mediated translocation of the insulin-sensitive glucose transporter, GLUT4, from cytoplasm to the plasma membrane of adipocytes. MAs^III^, and particularly DMAs^III ^is unstable in human urine [10,11] Therefore, analysis of these metabolites requires a special sample handling and optimized analytical techniques. Indeed, both MAs^III ^and DMAs^III ^were detected in urines collected from individuals exposed to iAs in drinking water and from acute promyelocytic leukemia patients undergoing arsenic trioxide treatment [12-17]. It has been hypothesized that because of their exceptional toxicities, MAs^III ^and DMAs^III ^contribute to the adverse effects of iAs exposure. Results of numerous laboratory studies support this hypothesis [18-20]. However, to date only one study examined association between the production of MAs^III ^and DMAs^III ^and the adverse effects of iAs exposure in humans. This study by Valenzuela and associates showed that carriers of AS3MT/287Thr polymorph who have higher concentrations of MAs^III ^in urine could be more susceptible to adverse effects of iAs exposure than carriers of wild-type AS3MT (AS3MT/287Met) [21].

The main goals of our study were (i) to reexamine the link between chronic exposure to iAs and diabetes, using multiple diabetes and exposure indicators, and (ii) to characterize the role of iAs metabolism, including MAs^III ^and DMAs^III ^production, in development of diabetes by individuals exposed to iAs in drinking water. Results of our work show that risk of diabetes significantly increases with iAs exposure and that urinary DMAs^III ^is positively associated with this risk.

## Methods

### Study Subjects and Sample Collection

Study subjects were recruited among residents of the Zimapán and Lagunera regions (Mexico), using information on current and historical levels of iAs in local drinking water supplies [22-26]. Potential participants who gave written informed consent were invited for an interview during which they completed a detailed questionnaire focusing on residence in the study areas, use and sources of drinking water, history of disease, and potential occupational exposures to As. The subjects with a minimum of two-year residency in the Zimapán or Lagunera regions and without occupational exposures to As were recruited for the study. Pregnant women, alcoholics, and individuals with chronic or acute diseases of the urinary tract or with type I diabetes were excluded from this study. In order to facilitate future analyses of the genotype-phenotype associations, we recruited only genetically unrelated individuals. All procedures included in this study were approved by Institutional Review Boards of Cinvestav-IPN, Mexico City, Mexico and University of North Carolina at Chapel Hill, North Carolina, USA.

All subjects were examined by an experienced physician and body weight, height, blood pressure, body mass index (BMI), and waist-to-hip ratio were recorded. A spot urine sample was collected from each subject during the medical exam. The urines were aliquoted and snap-frozen in dry ice immediately after collection. Each subject also provided a sample of water he or she had typically used for drinking. For diagnosis of diabetes, a sample of fasting blood was collected into EDTA-vacutainers (Becton, Dickinson and Co., Franklin Lakes, NJ), followed by oral glucose tolerance test (OGTT). Here, each subject drank a glass of water (~200 mL) containing 75 g of anhydrous glucose (Mallinckrodt Baker, Inc., Xalostoc Mexico). A sample of venous blood was collected two hours after glucose administration. Plasma was prepared from both fasting and two-hour blood samples and stored at -80°C.

### Analyses of As in Urine and Drinking Water

**Figure 1:**
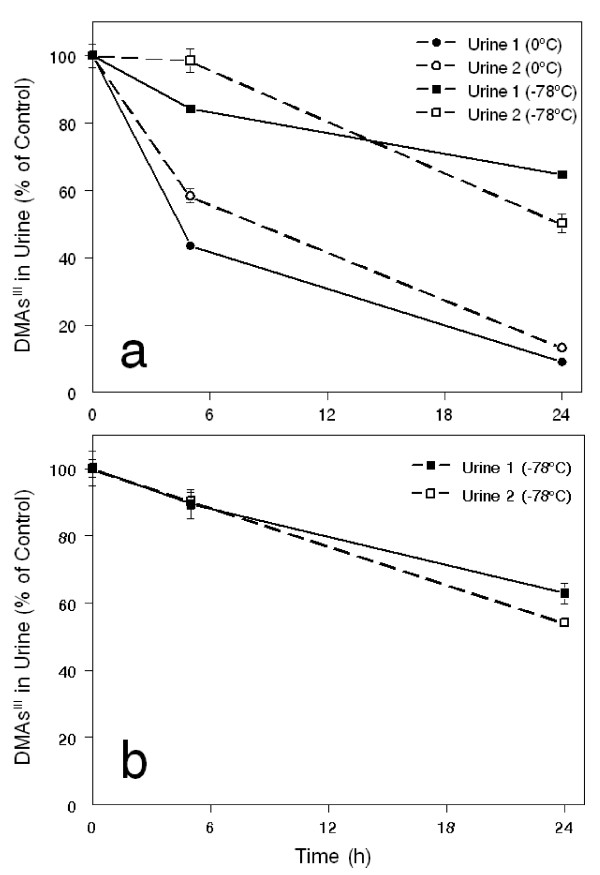
**Effect of the storage time and temperature on DMAs^III ^oxidation in human urine**. Spot urines from two subjects were spiked with iododimethylarsine (DMAs^III^I) at final concentrations of 15 ppb As (a) or 100 ppb As (b). DMAs^III ^contents were determined in urines immediately after spiking (Control) and after 6 and 24 hours after spiking in urines stored either on ice (0°C) or in dry ice (-78°C). Mean ± SD are shown for n = 3.

**Figure 2:**
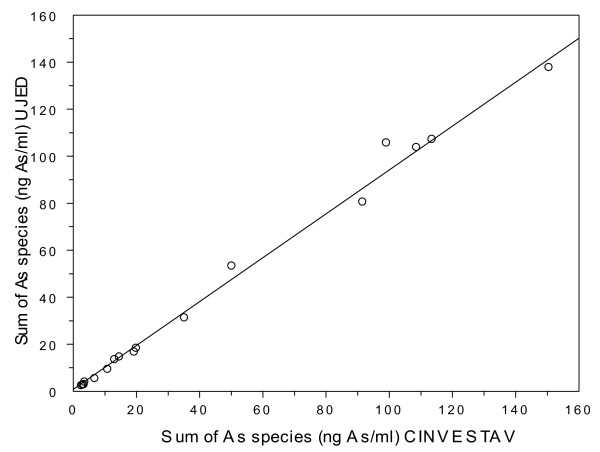
**Correlation between sums of As species in urines of eighteen Zimapán residents analyzed in both Cinvestav-IPN and in Universidad Juárez del Estado de Durango (UJED): R = 0.997, p < 0.01)**.

Only six samples of urine were collected in one day to facilitate quantitative analysis of the unstable MAs^III ^and DMAs^III^. Samples collected in Zimapán were packed in dry ice and transported by car to Cinvestav-IPN in Mexico City (a two-three hour drive from Zimapán). In the Lagunera region, urines were collected in Universidad Juárez del Estado de Durango (UJED) and analyzed in a local laboratory. The analysis of trivalent arsenicals, including MAs^III ^and DMAs^III^, was completed within 6 hours after collection. Our tests show that on average only about 9% of DMAs^III ^in urine oxidizes under these conditions (Figure 1). Pentavalent arsenicals were analyzed later in aliquots of urine that were stored at -80°C. The metabolites of iAs in urines were analyzed by hydride generation (HG)-atomic absorption spectrometry (AAS) with cryotrapping (CT). This method has been optimized to detect all tri- and pentavalent metabolites of iAs except dimethylthioarsinic acid [27,28]. Laboratory in Cinvestav-IPN used a traditional HG-CT-AAS system with a conventional quartz tube atomizer [28]; a more advanced HG-CT-AAS system equipped with multiatomizer was used in UJED. Calibration curves (0.5, 1, 2, 3, and 4 ng As/mL) were prepared using tri- and pentavalent As standards as previously described [28]. The Urine Arsenic reference materials (Laboratoire de Toxicologie, INSPQ, Québec, Canada) that contain 25.1 (S0907), 67.0 (S0902), and 202.5 ng As/mL (S0906) were used for quality control. The concentrations of As species in urine were expressed as ng As/mL or as μg As/mg creatinine. The concentrations of creatinine were measured by Jaffe reaction (Randox Laboratories Ltd., San Diego, CA). To control for possible differences in precision between the two analytical laboratories, a set of eighteen urines collected in Zimapán and analyzed in Cinvestav-IPN were re-analyzed in UJED. A nearly perfect correlation (R = 0.997, p < 0.01) was found for the 2 sets of results, specifically for the sums of As species detected in the urines (Figure 2).

The concentrations of iAs in drinking water and concentrations of total As (tAs) in urines were determined by HG-atomic fluorescence spectrometry (HG-AFS) as previously described [27,29]. The Trace Elements in Water standard reference material (SRM 1643e, National Institute of Standards and Technology, Gaithersburg, MD) containing 60.4 ± 0.7 ng As/mL was used for quality control. The concentration of iAs in water were expressed as ng As/mL or ppb; tAs concentration in urine were expressed as ng As/mL or as μg As/mg creatinine.

### Analyses in Blood and Plasma

1. Glucose concentrations were determined in the fasting and 2-hour blood by HemoCue Glucose 201RT glucometer (HemoCue, Lake Forest, CA) that reports results in plasma glucose equivalents.

2. Concentration of glycated hemoglobin HbA1c was measured in fasting blood, using a GDX A1c test cartridges (Cholestech Corp., Hayward, CA).

3. Insulin concentration was determined in fasting blood plasma using the Human Insulin ELISA Kit (Linco Research, Inc., St. Charles, MO).

### Evaluation of iAs Exposure and Metabolism

The following indicators were used to estimate individual exposure to iAs in drinking water:

1. The concentration of iAs in drinking water samples provided by study subjects.

2. The concentration of tAs in spot urines collected during medical exams.

3. Cumulative exposure estimated from measurements of current and historical concentrations of iAs in drinking water supplies in Zimapán and Lagunera and information from the questionnaires about each subject's length of residence in these areas [22,25,26]. The measurements of iAs concentration were used to estimate the average annual exposure at each location in the study area during the years 1993 to 2008. When a location had no measurements in a given year, the concentration of iAs was assumed to have remained constant since the last year for which data was available. Cumulative exposure in ppm.years was estimated for each subject by summing the annual exposures over the years during which the subject had lived in the study area. Cumulative exposure could not be estimated for the period before 1993 because only scattered measurements for a few locations were available from 1978 to 1992 and no data were available for 1977 and before. Estimates of cumulative exposures were therefore generated for the 1993-2008 period and, to examine temporal effects, for the 5-year segments 1993-1997, 1998-2002 and 2003-2007.

The metabolic patterns for iAs were characterized by the concentration or relative amount (% of tAs) of each of the tri- and pentavalent metabolites of iAs in urine and by the ratios of DMAs/MAs, MAs/iAs, and (DMAs+MAs)/iAs.

### Classification of Diabetes Status

Diabetic individuals were identified by fasting blood glucose level (FBG) ≥126 mg/dL, by the two-hour blood glucose level (2HBG) ≥200 mg/dL determined by OGTT, and by self-reported doctor diagnosis of diabetes or use of anti-diabetic medication as indicated in the questionnaires. Individuals who reported previous diagnoses of diabetes or taking diabetes medication were not administered OGTT. Classification by 2HBG and FBG gave nearly identical results, so in the text we report results for both indicators combined. Results for separate analyses based on FBG and 2HBG are given in Additional Material. Fasting plasma insulin (FPI) and homeostasis model assessment - insulin resistance (HOMA-IR = FPI [μU/mL] × FBG [mmol/L]/22.5) were used to characterize insulin production and insulin resistance.

### Statistical Analysis

Standard descriptive analyses were carried out using means and standard deviations for continuous variables and frequencies for categorical variables. The distributions of continuous variables were examined graphically using histograms and normal probability plots. Measurements of iAs in water and most biological indicators, including iAs and its metabolites in urine were approximately log-normally distributed and for these we report the geometric mean and standard deviation. Measured values of MAs^III ^and DMAs^III ^that were below the limit of detection (LOD) were replaced with LOD/2^1/2 ^for statistical analysis. The LOD for both metabolites was 0.1 ng/mL As. Associations of diabetes status with iAs and urinary metabolites of iAs were examined using logistic regression to estimate odds ratios (ORs) and 95% confidence intervals (CIs). iAs concentration in drinking water and concentrations of tAs and iAs metabolites in urine were examined both as continuous variables and in categorical form. To control for potential confounding, year of age, sex, hypertension (systolic blood pressure ≥140, diastolic blood pressure ≥90 or reported use of anti-hypertensive drugs) and obesity (BMI > 30) were included as covariates. Although the effects of adjustment were negligible (< 10% change in the OR), all covariates were retained in the models because of their known association with diabetes. Associations of continuous variables for 2HBG, FBG, FPI, and HOMA-IR with measures of tAs and iAs metabolites were analyzed by linear regression, with log-transformation of tAs in water and of tAs and iAs metabolites in urine. Age, sex, hypertension and obesity, classified as described above, were included as covariates in these models. The assumption of linear exposure-response was assessed graphically and by fitting alternative models with flexible spline functions and was found to be adequate. Because of the different concentration ranges of urinary metabolites, we report ORs, regression coefficients, and CIs for those indicators for a one interquartile range (IQR) increment of exposure to facilitate comparison. Analyses of urinary metabolites of iAs were conducted both with and without urinary creatinine concentration as a covariate to assess possible effects of variation in urine volume. Statistical adjustment for creatinine concentrations changed the numerical values of some regression coefficients, but the interpretation and statistical significance of the findings remained the same. Statistical analyses reported here were performed in Stata/MP version 11.2 for Mac OS × (StataCorp, College Station, TX).

## Results

### Characteristics of the Study Population and Exposure to iAs

We recruited a total of 258 subjects, including 147 Zimapán and 111 Lagunera residents, both females (109 and 65, respectively) and males, five years old or older. Seventy-three subjects were 18 years old or younger. Sixty subjects were fifty years old or older. One hundred and nine subjects reported using only tap (municipal) water or water from private wells for both drinking and cooking. Twenty subjects drank both tap and bottled water, but used only tap water for cooking. Twenty subjects drank only bottled water, but used the tap or well water for cooking. Ninety one subjects, mostly from Zimapán, used only bottled water for both drinking and cooking. One subject used only water from a local spring. Basic characteristics of the study population are provided in Table 1.

**Table 1:** Descriptive characteristics of the study population

	Diabetic		Non-diabetic	
	
	Mean (N)	SD (%)	Mean (N)	SD (%)
Population (N, %)	(25)	(9.7)	(233)	(90.3)

Female (N, %)	(19)	(76.0)	(155)	(66.5)

Age (years)	50.4	13.1	32.3	18.0

iAs concentration in drinking water (ppb)	77.3	61.8	39.2	44.9

Water consumption (L/day)	2.1	1.0	1.9	0.9

Bottled water for drinking (N, %)	(12)	(48.0)	(132)	(56.7)

BMI > 30 (N, %)	(11)	(44.0)	(77)	(33.1)

Hypertensive (N, %)	(9)	(36.0)	(88)	(37.8)

FBG (mg/dL)	177.4	77.5	83.8	16.0

2HBG (mg/dL)	258.4	130.0	97.3	27.4

HbA1c (%)	9.0	2.6	6.1	0.6

FPI	7.8	6.4	9.0	6.3

HOMA-IR	3.1	2.3	1.8	1.3

**Figure 3:**
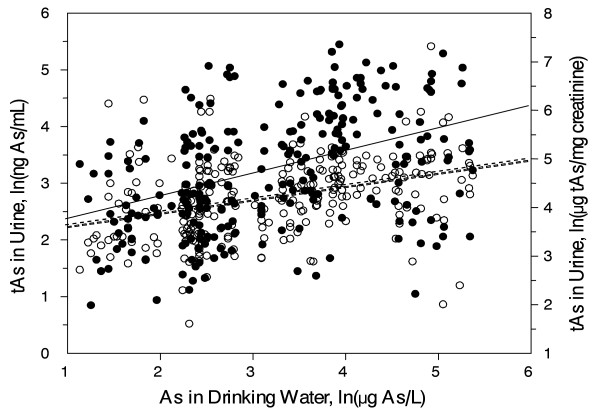
**Correlations between logarithmically transformed concentrations of iAs in drinking water and tAs in urines collected from subjects in Zimapán and Lagunera: ● tAs in ng/mL (full line); ○ tAs in μg/mg of creatinine (dashed line)**.

The concentrations of iAs in samples of drinking water ranged from 3 to 215 ppb (ng As/mL) (Table 2). The concentrations of tAs in urine ranged from 2 to 234 ng As/mL (5 to 1,512 ng As/mg creatinine). The levels of tAs determined in the chemically digested urines correlated strongly with the sum of tri- and pentavalent metabolites of iAs determined by HG-CT-AAS (R = 0.98; p < 0.01). Sum of iAs metabolites in urine represented 95.2 ± 12.2% (mean ± SD) of tAs. These data suggest that iAs metabolites accounted for most of As in urines and that urines did not contain significant amounts of thioarsenicals or complex organic As species that cannot be detected by HG-CT-AAS. We found a statistically significant correlation between the levels of iAs in drinking water and tAs in urine (R = 0.27; p < 0.01) (Additional file 1). This correlation was stronger for the logarithmically transformed levels of iAs and tAs, both before and after normalization for creatinine: R = 0.42 (p < 0.01) and R = 0.36 (p < 0.01), respectively (Figure 3).

**Table 2:** Descriptive statistics for exposure to iAs in drinking water and iAs metabolites in urine

*Exposure to iAs in Water*	**N**^**a**^	Range		AM	GM	GSD
iAs, current concentration (ppb)	258	3.1	215.2	42.9	24.4	2.9

iAs, cumulative exposure 1993-2008 (ppm.years)	255	0.0	6.73	1.8	1.3	2.3

iAs, cumulative exposure 1993-1997 (ppm.years)	255	0.0	5.8	0.7	0.5	2.8

iAs, cumulative exposure 1998-2002 (ppm.years)	255	0.0	4.5	0.6	0.5	2.3

iAs, cumulative exposure 2002-2007 (ppm.years)	255	0.0	1.2	0.5	0.4	2.1

***Metabolites of iAs in Urine (ng/mL)***						

tAs	256	2.3	233.7	41.2	24.7	2.8

iAs^III ^	257	0.1	37.2	4.2	2.1	3.4

MAs^III ^	257	ND	2.4	0.4	0.1	2.3

DMAs^III^	257	ND	64.8	4.7	0.9	3.2

iAs^V^	257	ND	32.9	1.4	1.5	4.7

MAs^V ^	257	0.1	36.2	5.0	1.1	1.1

DMAs^V^	257	0.1	162.6	22.5	2.4	1.3

DMAs/MAs ratio	257	0.4	26.4	5.6	4.8	1.7

MAs/iAs ratio	257	0.0	5.1	1.3	1.1	2.0

Abbreviations: AM, arithmetic mean; GM, geometric mean; GSD, geometric standard deviation; ND, not detected.

^a ^All 258 subjects provided sample of drinking water for iAs analysis; urine samples were collected from 257 subjects and analyzed for As species; only 256 urine samples were analyzed for tAs; cumulative exposure was calculated for 255 subjects.

### Urinary Metabolites of iAs

Most of the urine samples contained both tri- and pentavalent metabolites of iAs, including iAs^III^, iAs^V^, MAs^III^, MAs^V^, DMAs^III^, and DMAs^V ^(Table 2). TMAsO was not detected. MAs^III ^was detected in 67% of urines collected in Zimapán and 100% of urines collected in Lagunera; DMAs^III ^was detected in 75% and 100% of urines collected in Zimapán and Lagunera, respectively. In these urines, MAs^III ^represented 0.1% to 18% (median 0.9%) and DMAs^III ^0.2% to 81% (median 8.6%) of tAs. We found no statistically significant associations between the concentration or percentage of DMAs^III ^or MAs^III ^in urine and either age or gender of the study subjects. However, the young (≤18-year old) subjects had a lower average DMAs/MAs ratio (4.7) than either subjects older than 18 years (5.9, p < 0.01) or subjects older than 50 years (6.1, p < 0.01). We also found statistically significant positive correlations (p < 0.01) between DMAs/MAs ratio and iAs exposure represented by either iAs concentration in drinking water or tAs concentration in urine. In contrast, the MAs/iAs ratio was negatively associated with iAs concentration in drinking water (R = -0.19, p < 0.01) and tAs concentration in urine (R = -0.22, p < 0.01).

### Associations of Diabetes with iAs Exposure and Metabolism

Distribution of FBG, 2HBG, and HbA1c values among study subjects is shown in Table 1. As expected, we found strong positive correlations (R ≥ 0.78; p < 0.01) between these three diabetes indicators. All subjects with FBG ≥126 mg/dL or 2HBG ≥200 mg/dL were thirty years old or older. Similarly, abnormally high HbA1c levels (> 7%) were found almost exclusively in subjects thirty five years old or older. Diabetes classified by the combination of FBG, 2HBG and reported diagnosis or medication was positively associated with the concentration of iAs in drinking water (Table 3 and Additional files 2 and 3), with the OR increasing 13% per 10 ppb of iAs (95% CI 1.05-1.22, p < 0.01) for the combined classification (OR 1.15, 95% CI 1.06-1.24, p < 0.01 for classification by FBG, OR 1.14, 95% CI 1.06-1.24, p < 0.01 for classification by 2HBG). Diabetes was positively associated with cumulative exposure to iAs during most recent five-year window but the OR was imprecise, and there was no association with overall cumulative exposure in the 1993-2008 period or with exposure in earlier five-year windows (Table 3).

**Table 3:** Association of diabetes^a ^with exposure to iAs in drinking water and iAs metabolites in urine, adjusted for age, sex, obesity and hypertension

*Exposure to iAs in Water*	**OR**^**b**^	95% CI		**p**^**c**^
iAs, current concentration	1.13	1.05	1.22	< 0.01

iAs, cumulative exposure 1993-2008	1.03	0.77	1.39	0.83

iAs, cumulative exposure, 2003-2007	3.57	0.90	14.19	0.07

iAs, cumulative exposure, 1998-2002	0.98	0.41	2.37	0.97

iAs, cumulative exposure, 1993-1997	0.88	0.52	1.48	0.61

***Metabolites of iAs in Urine***				

tAs	1.12	0.78	1.62	0.54

iAs^III ^	0.94	0.61	1.46	0.79

MAs^III ^	1.24	0.60	2.54	0.35

DMAs^III^	1.24	1.00	1.55	0.05

iAs^V ^	1.06	0.94	1.18	0.35

MAs^V ^	0.92	0.58	1.43	0.70

DMAs^V^	0.97	0.66	1.46	0.87

DMAs/MAs ratio	1.38	0.90	2.13	0.13

MAs/iAs ratio	1.02	0.92	1.13	0.72

Abbreviations: OR, odds ratio; CI, confidence interval.

^a ^Diabetes classified by either fasting blood glucose ≥126 mg/dL, 2-hour blood glucose ≥200 mg/dL, self-report of doctor diagnosis or use of anti-diabetic medication.

^b ^Units of OR and CI are 10 ppb for iAs concentration and ppm.years for cumulative iAs exposure. ORs for iAs metabolites are standardized to an increment of one inter-quartile range.

^c ^p-value for comparison of cases to individuals free of diabetes.

Diabetes was not notably associated with tAs or with most of the metabolites of iAs in urine (Table 3). However, a marginally significant association (OR 1.24, 95% CI 1.00-1.55, p = 0.05) was found with the urinary concentration of DMAs^III^. The association of diabetes with urinary DMAs^III ^was statistically significant (p = 0.04) when diabetes was classified only by 2HBG≥200 and reported diagnosis or medication, but and on the borderline of significance (p = 0.05) when FBG≥126 mg/dL was used (Additional file 4). The OR for diabetes increased slightly after controlling for creatinine (OR 1.37 per IQR 95% CI 1.10-1.72, p = 0.01, data not shown).

HbA1c level in blood was positively associated with iAs in drinking water (p = 0.03) but unrelated to iAs or its metabolites in urine (Table 4). FPI and HOMA-IR, which characterize insulin production and resistance, respectively, were negatively associated with the levels of iAs in drinking water (p < 0.01) and with the concentrations of tAs, iAs^III ^and DMAs^III ^in urine (p ≤ 0.01) (Table 4). FPI was also negatively associated with urinary MAs^III ^(p = 0.02).

**Table 4:** Association of log-transformed HbA1c, FPI and HOMA-IR with log-transformed concentrations of iAs in drinking water and of iAs metabolites in urine, adjusted for age, sex, obesity and hypertension

	HbA1c					FPI		HOMA-IR				
	**β**^**a**^	**95% CI**		**p**^**b**^	**β**^**a**^	**95% CI**		**p**^**b**^	**β**^**a**^	**95% CI**		**p**^**b**^

Water iAs	0.193	0.018	0.369	0.03	-2.084	-2.720	-1.448	< 0.01	-1.641	-2.358	-0.924	< 0.01

Urinary tAs	0.164	-0.570	0.898	0.66	-5.313	-8.068	-2.559	< 0.01	-4.538	-7.514	-1.562	< 0.01

Urinary iAs^III ^	-0.003	-0.075	0.069	0.94	-0.534	-0.803	-0.266	< 0.01	-0.409	-0.698	-0.121	< 0.01

Urinary MAs^III ^	0.003	-0.031	0.078	0.39	-0.037	-0.608	-0.007	0.02	-0.020	-0.053	0.013	0.25

Urinary DMAs^III^	0.023	-0.031	0.078	0.40	-0.404	-0.608	-0.199	< 0.01	-0.301	-0.523	-0.078	< 0.01

Urinary iAs^V ^	0.008	-0.005	0.021	0.23	-0.028	-0.079	0.023	0.29	-0.041	-0.096	0.014	0.14

Urinary MAs^V ^	0.007	-0.086	0.100	0.88	-0.421	-0.777	-0.065	0.02	-0.292	-0.670	0.087	0.13

Urinary DMAs^V^	0.085	-0.281	0.452	0.65	-1.282	-2.689	0.125	0.07	-0.884	-2.382	0.615	0.25

Urinary DMAs/MAs ratio	0.074	-0.043	0.192	0.22	-0.403	-0.856	0.050	0.08	-0.372	-0.859	0.114	0.13

Urinary MAs/iAs ratio	0.007	-0.016	0.030	0.56	0.092	0.003	0.182	0.04	0.123	0.030	0.216	0.01

Abbreviations: HbA1c, glycated hemoglobin; FPI, fasting plasma insulin; HOMA-IR, homeostasis model assessment - insulin resistance; β, regression coefficient; CI, confidence interval.

^a ^Units of β and CI are 10 ppb for iAs concentration. Coefficients for metabolites are standardized to an increment of one inter-quartile range.

^b ^p-value for test of β = 0.

## Discussion

The arsenicosis-endemic areas of Zimapán and Lagunera were chosen for this study because tens of thousands of local residents are currently exposed to a wide range of iAs concentrations in drinking water and because relatively detailed information exists about historical levels of iAs in the local drinking water supplies. Unlike most previous studies, we used multiple biomarkers to classify diabetes and to characterize exposure to iAs. In spite of a relatively small number of subjects recruited for this study we were able to show that the exposure to iAs was positively associated with all three diabetes indicators. Our findings contradict results of a recent study that found no significant association of iAs exposure with diabetes in another arsenicosis area located in Bangladesh [30]. This study used only glycated hemoglobin (HbA1c) and glucosuria to classify diabetic individuals. Although the HbA1c level in blood is now an accepted measure for diagnosis of diabetes in the US, it is unclear whether it was also validated for the Bangladeshi population. Glucosuria is not a reliable indicator of diabetes [31]. Thus, using the validated diabetes indicators may be essential for linking iAs exposure to risk of diabetes. Notably, our data on the negative associations between iAs exposure and FPI and HOMA-IR suggest that the mechanisms of iAs-induced diabetes differ from those underlying type-2 diabetes, which is typically characterized by insulin resistance (i.e., increased HOMA-IR) and hyperinsulinemia [32].

In our study, FPG and 2HBG were significantly associated with iAs concentration in drinking water, but not with tAs or sum of iAs metabolites in urine. Samples of water provided for iAs analysis represented the types and sources of drinking water that were used by study subjects on a daily basis for an extensive period of time. Thus, it is plausible that iAs concentrations in these samples are more representative of the current exposures to iAs than are tAs levels in spot urines, which could be affected by changes in drinking water sources or consumption during days or even hours before urine collection. Our results show that diabetes was not associated with cumulative exposure to iAs over the last fifteen years. Historical records show that levels of iAs in drinking water supplies in both Zimapán and Lagunera areas have changed in recent years as a result of interventions by local governments. These changes may explain the low correlation between estimated cumulative exposure and the current concentrations of iAs in drinking water. In addition, the use of bottled water for drinking and cooking has become common, particularly in Zimapán, which historically had the higher levels of iAs in the municipal water supplies.

Previous epidemiologic studies used MAs/iAs and DMAs/MAs ratios in urine to evaluate the efficiency of iAs methylation and detoxification by subjects exposed to environmental iAs. Some of these studies reported a negative association between iAs exposure and the DMAs/MAs ratio or %DMA in urine, suggesting saturation or inhibition of the methylation pathway for iAs at high exposure levels [33]. Other reports have linked low DMAs/MAs ratio (or high MAs/DMAs ratio) and high %MAs (or low %DMAs) to an increased susceptibility to diseases associated with iAs exposure, including skin lesions [34-36]. However, our data suggest that with increasing iAs exposure the efficiency of iAs methylation increases as indicated by higher DMAs/MAs and lower MAs/iAs ratios in urine.

An important goal of the present study was to examine associations between the prevalence of diabetes and urinary concentrations of MAs^III ^and DMAs^III^. All urine samples collected in Lagunera and analyzed in UJED by the advanced HG-CT-AAS system contained both MAs^III ^and DMAs^III^. The failure to detect DMAs^III ^and, particularly MAs^III ^in a large number of urines from Zimapán was likely due to a lower sensitivity of the conventional HG-CT-AAS system used in Cinvestav-IPN. Notably, DMAs^III ^in urine was associated positively with FBG and 2HBG but negatively with FPI. These results suggest that individuals with high concentrations of DMAs^III ^in urine are at increased risk of developing diabetes. DMAs^III ^is a potent inhibitor of insulin-stimulated glucose uptake by adipocytes [8]. DMAs^III ^also inhibits glucose-stimulated insulin secretion by isolated murine pancreatic islets (Styblo and Douillet, unpublished data). Thus, the mechanism of the diabetogenic effects of iAs exposure may involve inhibition by DMAs^III ^of insulin-dependent glucose uptake and metabolism in peripheral tissues and/or inhibition by DMAs^III ^of insulin production by pancreatic β-cells. Taken together these two mechanisms would produce fasting hyperglycemia and impaired glucose tolerance, as well as decreased FPI and HOMA-IR values, i.e., symptoms that are consistent with results of our study.

While this study had notable strengths, the interpretation of the results may be affected by several limitations. The study population was predominantly female because men in the area often emigrate to work. Although we adjusted for sex in the analysis, the ability to extend the findings to populations with a more balanced sex ratio may be limited to the extent that women and men may differ in susceptibility to As-related disease. As noted above, iAs exposures in the study area have changed in recent years as a result of modifications to municipal water systems and the use of bottled water. The negative correlation between current and cumulative exposures suggests that areas with historically high iAs levels were targeted for interventions to reduce exposure. Our data (not shown) suggest that individuals who had been told by a doctor that they had diabetes were not more likely to use bottled water, however. The recent changes in exposure add to the challenges of determining whether measurements in drinking water versus urine or current versus cumulative exposure are the best measure of biologically-relevant exposure to iAs. Although we were able to estimate exposure from 1993 onward, 64% of the subjects had lived in the study area before 1993 and had longer exposure histories than we could estimate. The sparse environmental data from the 1980s suggest that iAs levels in municipal water supplies may have been higher in that era than in later years. No measurements were available for years before 1978, but it is possible that iAs exposures were lower before the construction of municipal water systems served by deep wells began in the 1960s. In summary, there are plausible mechanisms for errors resulting in both under- and over-estimation of cumulative iAs exposure, but the magnitude and direction of bias in exposure-disease associations as a result of any such errors cannot be assessed without additional data.

The American Diabetes Association has recommended that FPG, OGTT or HbA1c be used to test for diabetes or to assess risk of future diabetes in asymptomatic patients [37]. However, there is a potential for misclassification of diabetes status due to error in the field methods we used to measure these indicators. Any such error is likely to be random with respect to arsenic exposure, and so most likely to attenuate associations between diabetes status and exposure. We also lacked reliable indicators of social class and were consequently unable to adjust for it in the analysis. Both Zimapan and Lagunera are rural, low income areas of Mexico. Recent data suggest that type-2 diabetes is less prevalent among Mexican residents with low socioeconomic status [38]. Nevertheless, the potential magnitude of confounding by social class is difficult to assess, because of the limited information about the extent to which it is a risk factor for diabetes in rural Mexican populations.

Finally, the cross-sectional design has well known limitations. In the present study, these relate primarily to the inability to measure the changes in exposure discussed above. Because we did not know when the onset of diabetes occurred, cumulative exposures may also have been overestimated for subjects who had the disease for a number of years. Such overestimation would tend to understate the association of exposure with disease. Cross-sectional studies can be susceptible to selection bias if exposed individuals withdraw from the population because of exposure or its early effects. However, exposure to iAs in drinking water was not recognized as a health concern until recently, so it is unlikely to have prompted more highly exposed individuals to move out of the study area. In addition, individuals with prevalent and new-onset diabetes may differ in the way they metabolize arsenic.

## Conclusions

Our research links exposure to iAs in drinking water to an increased risk of developing diabetes which is characterized by fasting hyperglycemia and impaired glucose tolerance. However, unlike typical type-2 diabetes, iAs-related diabetes was not associated in this study with increased insulin resistance as measured by HOMA-IR. In addition, low FPI levels seem to suggest that β-cell function may be impaired by exposure to iAs or its toxic methylated metabolites. In summary, we believe that strengths of our research outweigh the above-described limitations, providing additional evidence about the diabetogenic effects of iAs exposure.

## List of Abbreviations

2HBG: 2-hour blood glucose level determined by OGTT; AAS: atomic absorption spectrometry; AFS: atomic fluorescence spectrometry; ALT: alanine aminotransferase; As: arsenic; AS3MT: arsenic (+3 oxidation state) methyltransferase; AST: aspartate aminotransferase; BMI: body mass index; CI: confidence interval; CINVESTAV-IPN: Centro de Investigación y de Estudios Avanzados del Instituto Politécnico Nacional; CT: cryotrapping; DMAs: dimethylarsenic; DMAs^III^: dimethylarsinite; DMAs^III^I: iododimethylarsine; DMAs^V^: dimethylarsinite; FBG: fasting blood glucose; FPI: fasting plasma insulin; GLUT4: glucose transporter - type 4; HbA1c: glycated hemoglobin; HG: hydride generation; HOMA-IR: homeostasis model assessment - insulin resistance; iAs: inorganic arsenic; iAs^III^: arsenite; iAs^V^: arsenate; IQR: inter-quartile range; MAs: methyl-As; MAs^III^: methylarsonite; MAs^III^O: oxomethylarsine; MAs^V^: methylarsonate; OGTT: oral glucose tolerance test; OR: odds ratio; PKB: protein kinase-B; SRM: standard reference material.

## Competing interests

The authors declare that they have no competing interests.

## Authors' contributions

Authors contributed to the article as follows: LMDR coordinated work performed by the Mexican team in both the Zimapan and Lagunera regions and directed most of the chemical and biochemical analyses of samples collected in this study. GGV directed the field work in the Lagunera region and was responsible for analysis of As species in urine collected in this region. OLV and EHC conducted environmental and biological sampling and data collection. LCSP helped to supervise the field activities and conducted data collection. JMC tested the stability of the methylated trivalent arsenicals, specifically DMAs^III ^in human urines. ZD participated in designing the field studies and contributed to method development for collection of biological samples. DL conducted the statistical analyses and participated in writing the manuscript. MS, as the principal investigator for this project, coordinated the work by both the Mexican and American teams and was responsible for the manuscript preparation. All authors read and approved the final manuscript

## Supplementary Material

Additional file 1 **Table A1**. Correlations of iAs exposure indicators in water and urine. Click here for file

Additional file 2 **Table A2**. Association of diabetes classified by FBG ≥126 with exposure to iAs in drinking water, adjusted for age, sex, obesity and hypertension. Click here for file

Additional file 3 **Table A3**. Association of diabetes classified by 2HBG ≥200with exposure to iAs in drinking water, adjusted for age, sex, obesity and hypertension. Click here for file

Additional file 4 **Table A4**. Separate associations of diabetes status classified by fasting blood glucose and 2-hour blood glucose with exposure to iAs in drinking water and iAs metabolites in urine, adjusted for age, sex, obesity and hypertension. Click here for file
