# Supplementary material for: Exposure to arsenic in drinking water is associated with increased prevalence of diabetes: a cross-sectional study in the Zimapán and Lagunera regions in Mexico
Source: Environ Health. 2011 Aug 24;10:73. doi: 10.1186/1476-069X-10-73 (PMC3169452; doi:10.1186/1476-069X-10-73)
Supplement: Additional file 1 — Table A1. Correlations of iAs exposure indicators in water and urine. [file 1476-069X-10-73-S1.DOC]

**Additional File 1**

Table A1. Correlations of iAs exposure indicators in water and urine.

|  | **iAs**  **water** | | **tAs** | **iAs^III^** | **MAs^III^** | **DMAs^III^** | **iAs^V^** | **MAs^V^** | **DMAs^V^** |
| --- | --- | --- | --- | --- | --- | --- | --- | --- | --- |
| **iAs, water** | | 1 |  |  |  |  |  |  |  |
| **tAs** | | 0.27 | 1 |  |  |  |  |  |  |
| **iAs^III^** | | 0.13 | 0.77 | 1 |  |  |  |  |  |
| **MAs^III^** | | 0.08 | 0.67 | 0.80 | 1 |  |  |  |  |
| **DMAs^III^** | | 0.24 | 0.61 | 0.50 | 0.54 | 1 |  |  |  |
| **iAs^V^** | | 0.05 | 0.23 | 0.14 | 0.09 | 0.17 | 1 |  |  |
| **MAs^V^** | | 0.08 | 0.82 | 0.79 | 0.72 | 0.53 | 0.17 | 1 |  |
| **DMAs^V^** | | 0.19 | 0.91 | 0.67 | 0.54 | 0.35 | 0.11 | 0.71 | 1 |
