# Supplementary material for: Exposure to arsenic in drinking water is associated with increased prevalence of diabetes: a cross-sectional study in the Zimapán and Lagunera regions in Mexico
Source: Environ Health. 2011 Aug 24;10:73. doi: 10.1186/1476-069X-10-73 (PMC3169452; doi:10.1186/1476-069X-10-73)
Supplement: Additional file 2 — Table A2. Association of diabetes classified by FBG ≥126 with exposure to iAs in drinking water, adjusted for age, sex, obesity and hypertension. [file 1476-069X-10-73-S2.DOC]

**Additional File 2**

**Table A2**. Association of diabetes classified by FBG ≥126 with exposure to iAs in drinking water, adjusted for age, sex, obesity and hypertension.

| **Current iAs concentration in water (ppb)** | **Cases** | **Non-cases** | **OR** | **95% CI** | | **p^a^** |
| --- | --- | --- | --- | --- | --- | --- |
| **<10** | 1 | 53 | 1.00 |  |  |  |
| **10-49.9** | 7 | 125 | 2.56 | 0.30 | 21.77 | 0.40 |
| **50-124.9** | 9 | 41 | 7.09 | 0.82 | 61.61 | 0.07 |
| **>=125** | 6 | 16 | 15.79 | 1.69 | 147.63 | 0.02 |

Abbreviations: FBG, fasting blood glucose; OR, odds ratio; CI, confidence interval.

^a^p-value for comparison of cases to non-case
