# Supplementary material for: Exposure to arsenic in drinking water is associated with increased prevalence of diabetes: a cross-sectional study in the Zimapán and Lagunera regions in Mexico
Source: Environ Health. 2011 Aug 24;10:73. doi: 10.1186/1476-069X-10-73 (PMC3169452; doi:10.1186/1476-069X-10-73)
Supplement: Additional file 3 — Table A3. Association of diabetes classified by 2HBG ≥200with exposure to iAs in drinking water, adjusted for age, sex, obesity and hypertension. [file 1476-069X-10-73-S3.DOC]

**Additional File 3**

**Table A3**. Association of diabetes classified by 2HBG ≥200with exposure to iAs in drinking water, adjusted for age, sex, obesity and hypertension.

| Current iAs concentration in water (ppb) | Cases | Non-cases | OR | 95% CI | | p^a^ |
| --- | --- | --- | --- | --- | --- | --- |
| <10 | 2 | 52 | 1.00 |  |  |  |
| 10-49.9 | 7 | 125 | 0.77 | 0.18 | 3.29 | 0.84 |
| 50-124.9 | 9 | 41 | 2.51 | 0.57 | 11.00 | 0.16 |
| >=125 | 6 | 16 | 5.01 | 1.02 | 24.70 | 0.02 |

Abbreviations: 2HBG, 2-hour blood glucose; OR, odds ratio; CI, confidence interval.

^a^p-value for comparison of cases to non-case
