# Supplementary material for: Exposure to arsenic in drinking water is associated with increased prevalence of diabetes: a cross-sectional study in the Zimapán and Lagunera regions in Mexico
Source: Environ Health. 2011 Aug 24;10:73. doi: 10.1186/1476-069X-10-73 (PMC3169452; doi:10.1186/1476-069X-10-73)
Supplement: Additional file 4 — Table A4. Separate associations of diabetes status classified by fasting blood glucose and 2-hour blood glucose with exposure to iAs in drinking water and iAs metabolites in urine, adjusted for age, sex, obesity and hypertension. [file 1476-069X-10-73-S4.DOC]

**Additional File 4**

**Table A4. Separate associations of diabetes status classified by fasting blood glucose and 2-hour blood glucose with exposure to iAs in drinking water and iAs metabolites in urine, adjusted for age, sex, obesity and hypertension.**

|  | **FBG ≥126**^a^ | | | | | **2HBG ≥200**^b^ | | | |
| --- | --- | --- | --- | --- | --- | --- | --- | --- | --- |
|  | **OR**^c^ | | **95% CI** | | **p**^d^ | **OR**^c^ | **95% CI** | | **p**^d^ |
| ***Exposure to iAs in Water*** |  | |  |  |  |  |  |  |  |
| iAs, current concentration | 1.15 | 1.06 | | 1.24 | <0.01 | 1.14 | 1.05 | 1.24 | <0.01 |
| iAs, cumulative exposure 1993-2008 | 0.92 | 0.65 | | 1.30 | 0.64 | 1.06 | 0.78 | 1.43 | 0.70 |
| iAs, cumulative exposure, 2003-2007 | 2.61 | 0.62 | | 11.02 | 0.19 | 3.08 | 0.74 | 12.74 | 0.12 |
| iAs, cumulative exposure, 1998-2002 | 0.99 | 0.52 | | 1.88 | 0.97 | 1.14 | 0.48 | 2.67 | 0.79 |
| iAs, cumulative exposure, 1993-1997 | 0.86 | 0.59 | | 1.24 | 0.42 | 0.95 | 0.56 | 1.55 | 0.79 |
|  |  |  | |  |  |  |  |  |  |
| ***Metabolites of iAs in Urine*** |  |  | |  |  |  |  |  |  |
| tAs | 1.004 | 0.995 | | 1.014 | 0.40 | 1.003 | 0.994 | 1.013 | 0.50 |
| iAs^III^ | 0.995 | 0.903 | | 1.096 | 0.92 | 0.995 | 0.901 | 1.099 | 0.92 |
| MAs^III^ | 1.719 | 0.575 | | 5.141 | 0.35 | 1.749 | 0.574 | 5.327 | 0.34 |
| DMAs^III^ | 1.047 | 1.000 | | 1.097 | 0.05 | 1.051 | 1.002 | 1.101 | 0.04 |
| iAs^V^ | 1.058 | 0.956 | | 1.172 | 0.31 | 1.055 | 0.951 | 1.171 | 0.35 |
| MAs^V^ | 0.989 | 0.903 | | 1.083 | 0.81 | 0.988 | 0.902 | 1.082 | 0.79 |
| DMAs^V^ | 0.999 | 0.983 | | 1.015 | 0.90 | 1.000 | 0.985 | 1.016 | 0.18 |
| DMAs/MAs ratio | 1.105 | 0.981 | | 1.245 | 0.13 | 1.105 | 0.975 | 1.253 | 0.14 |
| MAs/iAs ratio | 0.627 | 0.307 | | 1.281 | 0.17 | 0.673 | 0.344 | 1.319 | 0.22 |

Abbreviations: FBG, fasting blood glucose; 2HBG, 2-hour blood glucose; OR, odds ratio; CI, confidence interval.

^a^Diabetes classified by FBG, diagnosis or medication.

^b^ Diabetes classified by 2HBG, diagnosis or medication.

^c^ Units of OR and CI are 10 ppb for iAs concentration, ppm.years for cumulative iAs exposure and ng As/mL urine for metabolites.

^d^ p-value for comparison of cases to individuals free of diabetes.
